# AI-RADS: Successes and challenges of a novel artificial intelligence curriculum for radiologists across different delivery formats

**DOI:** 10.3389/fmedt.2022.1007708

**Published:** 2023-01-04

**Authors:** Alexander L. Lindqwister, Saeed Hassanpour, Joshua Levy, Jessica M. Sin

**Affiliations:** ^1^ Department of Internal Medicine, California Pacific Medical Center, San Francisco, CA, United States; ^2^ Geisel School of Medicine at Dartmouth, Dartmouth College, Hanover, NH, United States; ^3^ Department of Pathology and Laboratory Medicine, Dartmouth Health, Lebanon, NH, United States; ^4^ Department of Dermatology, Dartmouth Health, Lebanon, NH, United States; ^5^ Department of Radiology, Dartmouth Health, Lebanon, NH, United States

**Keywords:** radiology - education, radiology, artificial intelligence, artificial intelligence education, residency, machine learning, teaching radiology, training resident

## Abstract

**Introduction:**

Artificial intelligence and data-driven predictive modeling have become increasingly common tools integrated in clinical practice, heralding a new chapter of medicine in the digital era. While these techniques are poised to affect nearly all aspects of medicine, medical education as an institution has languished behind; this has raised concerns that the current training infrastructure is not adequately preparing future physicians for this changing clinical landscape. Our institution attempted to ameliorate this by implementing a novel artificial intelligence in radiology curriculum, “AI-RADS,” in two different educational formats: a 7-month lecture series and a one-day workshop intensive.

**Methods:**

The curriculum was structured around foundational algorithms within artificial intelligence. As most residents have little computer science training, algorithms were initially presented as a series of simple observations around a relatable problem (e.g., fraud detection, movie recommendations, etc.). These observations were later re-framed to illustrate how a machine could apply the underlying concepts to perform clinically relevant tasks in the practice of radiology. Secondary lessons in basic computing, such as data representation/abstraction, were integrated as well. The lessons were ordered such that these algorithms were logical extensions of each other. The 7-month curriculum consisted of seven lectures paired with seven journal clubs, resulting in an AI-focused session every two weeks. The workshop consisted of six hours of content modified for the condensed format, with a final integrative activity.

**Results:**

Both formats of the AI-RADS curriculum were well received by learners, with the 7-month version and workshop garnering 9.8/10 and 4.3/5 ratings, respectively, for overall satisfaction. In both, there were increases in perceived understanding of artificial intelligence. In the 7-lecture course, 6/7 lectures achieved statistically significant (*P* < 0.02) differences, with the final lecture approaching significance (*P* = 0.07). In the one-day workshop, there was a significant increase in perceived understanding (*P* = 0.03).

**Conclusion:**

As artificial intelligence becomes further enmeshed in clinical practice, it will become critical for physicians to have a basic understanding of how these tools work. Our AI-RADS curriculum demonstrates that it is successful in increasing learner perceived understanding in both an extended and condensed format.

## Introduction

The radiology community has made it clear that artificial intelligence (AI) is both an inevitability within clinical practice and a necessary area of training for future physicians; applications of machine learning in radiology are already integrating themselves within picture archiving and communication systems (PACS) and voice recognition software, with a rapidly expansion of marketplace of commercially available AI tools for practicing radiologists (1–5) and an exponential increase in clinical trials utilizing machine learning (1). Yet despite this, graduate medical education has lagged in preparing trainees how to understand what these developments may entail (3, 4, 6, 7). Our institution originally attempted to ameliorate this by creating an AI curriculum for residents integrated into regularly scheduled didactic sessions. This pilot course, entitled “AI-RADS,” was successful in its longitudinal 7-month form and was one of the first of its kind in terms of artificial intelligence curricula specifically for radiology residents (8).

However, this expanded form was felt to be potentially cumbersome to the schedules of all learners. In response, a truncated version of the course was created: the seven-month curriculum was condensed into a one day, 7 h session. This manuscript serves to report the successes and challenges associated with concatenating an extended curriculum in artificial intelligence education for radiologists in a digital medium. At time of writing, this course is unique in terms of educational praxis and approach, as it introduces artificial intelligence concepts through fundamental algorithms in a way specifically designed for people with limited mathematics and computational science backgrounds through the lens of clinical radiology.

## Methods

This one-day intensive workshop was based off of the previously published artificial intelligence curriculum, AI-RADS, though modified for a more limited session in an entirely virtual environment.

**Figure 1:**
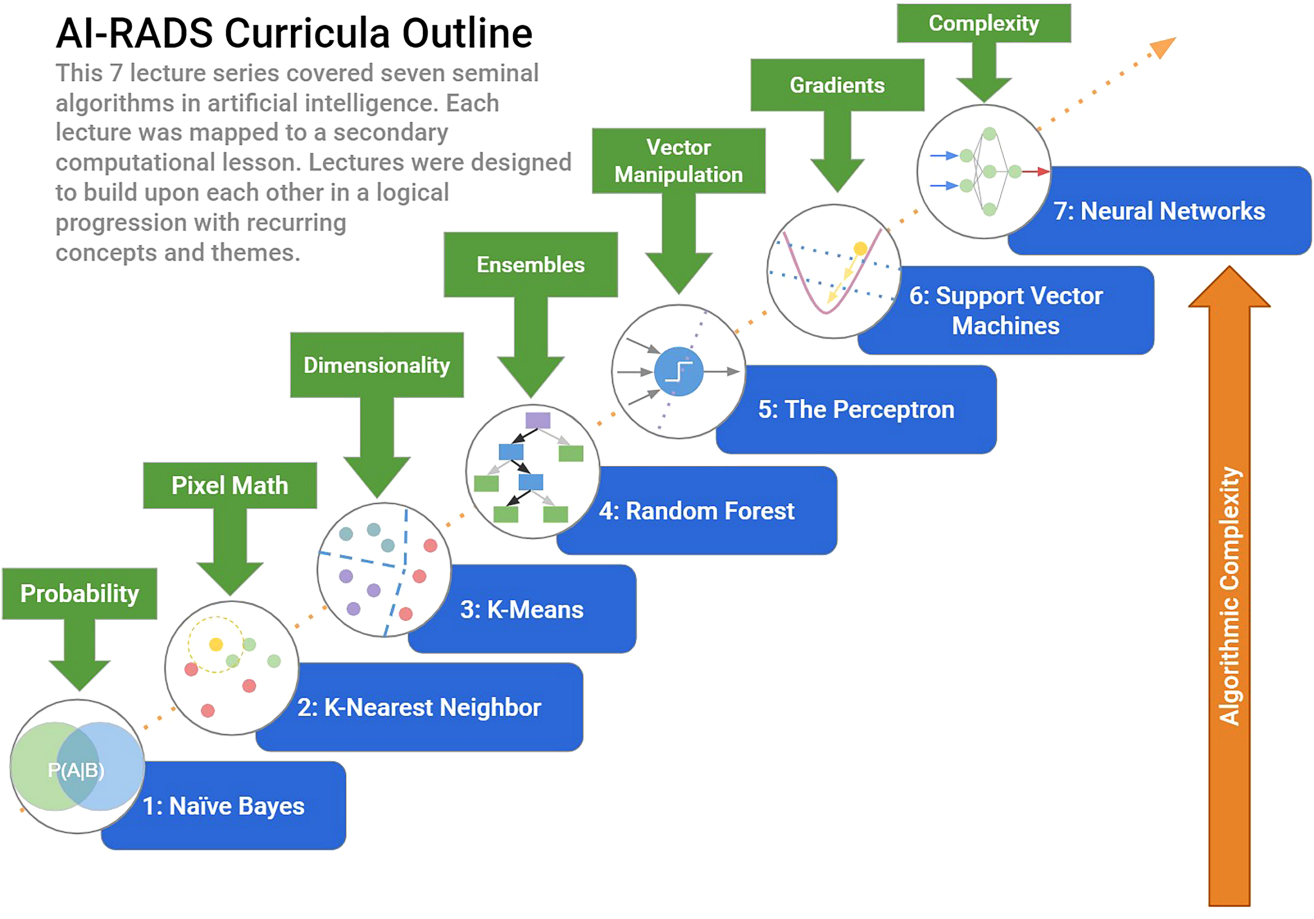
Longitudinal AI-RADS curriculum. The original AI-RADS Curriculum entailed a sequential progression of lectures based on seminal algorithms in artificial intelligence. Each lecture built on concepts introduced in the previous, with secondary computer science lessons integrated at each step.

The original curriculum consisted of seven lectures, with each lecture consisting of a fundamental algorithm in artificial intelligence. These algorithms were introduced as a string of simple observations about a common problem in modern computing, such as movie recommendations, spam filtering, etc. The goal of this approach was to cultivate an appreciation for the underlying simplicity rooted within some of these machine learning techniques, establish a sense of algorithmic thinking, and garner greater confidence in the learner's own understanding. Within each lecture, several secondary lessons in basic computing were incorporated such as pixel mathematics, data representation, and dimensionality. Lectures followed a cadence of increasing complexity and were presented as logical extensions of each other (Figure 1) (7). In the original course, lectures were accompanied by a journal club that would feature utilizing the previously discussed technique in action; this way direct sessions would be reinforced by practical examples in the primary literature to further draw clinical connections and reinforce the underlying material.

Performance metrics in the original course were attained through surveys administered before and after lectures. These included four content related questions that would ask attendees to rate their perceived ability to describe each topic on a Likert scale of 1 to 10. Attendees in the original AI-RADS course were residents from our home institution.

The condensed one-day version of the course was presented through a national radiologic society's monthly educational session. Due to ongoing constraints surrounding the pandemic, the workshop was administered virtually. Attendance was open to resident physician trainees at institutions within the geographic purview of the society; while attending physicians/physicians who have completed residency training were invited, their responses were not included in this analysis. Continuing medical education (CME) credit (up to 5.75 h) was offered for all participants.

**Figure 2:**
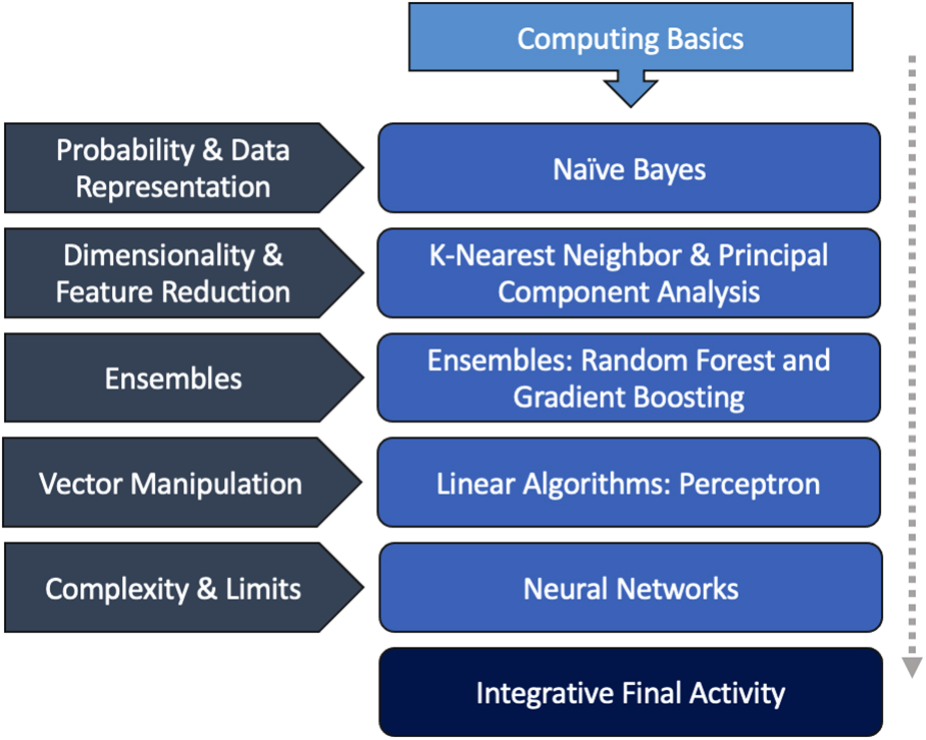
Intensive workshop AI-RADS curriculum. The outline of the one-day AI-RADS workshop followed a similar cadence and style as the original. A brief computing basics primer was introduced at the start of the course.Secondary themes in basic computer science and machine learning were incorporated into each didactic session. For some lectures, multiple algorithms were explored due to their similarity and relation to the secondary theme. The course ended in a final integrative activity that was meant to supplement the original curriculum's journal club.

The one-day workshop largely followed the same basic structure and organization as the longitudinal version of the course, though with some exceptions based on learner feedback on the original curriculum. Some of the more simplistic algorithms were removed in favor of expanding the explanations surrounding more difficult concepts, such as ensembles (Table 1). Basic computing terminology and concepts was frontloaded. All secondary computational lessons were preserved and integrated within the new lesson cadence (Figure 2), with a total content runtime of approximately six hours (see Appendix A). In the interest of time, algorithm specific readiness quizzes were substituted for a post session interactive activity. Metrics of quality were assessed *via* pre and post session surveys; all survey results were user-anonymous and entailed a combination of written and Likert-scale questions. Self-reported baseline level of familiarity with artificial intelligence was also collected (see Appendix B).

**Table 1:** Comparison of the 7 month and One-Day intensive AI-RADS curricula.

	7 Month Curriculum	One-day Intensive
Total Didactic Time	7 Hours	6 Hours
Algorithms Covered	7 (Naive Bayes, K-Nearest Neighbor, K-Means, Random Forest, The Perceptron, Support Vector Machines, Neural Networks)	7 (Naive Bayes, K-Nearest Neighbor, Principal Component Analysis, Random Forest, Gradient Boosting, The Perceptron, Neural Networks)
Delivery Method	In-Person	Virtual
Incorporation of Primary Literature	Biweekly 2 Hour Journal Clubs	Post-session Integrative Activity
Evaluation	Pre & Post Lecture Surveys	Pre & Post Session Survey

The AI-RADS curriculum contained several differences between delivery formats, as outlined above. Variations between algorithms covered were made based on learner feedback from the original seven month curriculum.

The integrative final activity represented an amalgamation of the previous course's journal clubs: participants were divided into groups, where each group was presented with a scenario and a description of a dataset (e.g., tasked to predict bone tumor diagnosis given a dataset of features). Groups were then instructed to discuss among themselves and select an algorithm explored in the workshop that would be best suited for each situation. Small groups would then report out their decision and a large group discussion would ensue, discussing potentially alternative selections as well as practical considerations of each approach. Each dataset and question were based on a real machine learning paper that employed one of the algorithms taught; the paper was revealed after the large group discussion. The goal of the integrative activity was to synthesize the techniques learned in a practical way and demonstrate that with even a basic understanding, radiologists without extensive AI backgrounds can effectively come up with the core components of published AI research.

For both the original course and the truncated workshop, all lectures and materials written and delivered by the medical student fellow in radiology. The student fellow had a degree in computational engineering and had previously written and instructed a course on computational biology for non-scientists as a graduate student before beginning his medical training.

### Statistical methods

Course demonstrations along with all figures attached were rendered using the Python 3 online shell, *Jupyter*. Content was reviewed by author SH, professor of computer science who specializes in AI. Survey information was analyzed using the statistical analysis package *SciPy* (version 1.8.1, 2022). Data distribution was assessed by calculating both skewness and kurtosis values; data was assumed to be unpaired given attrition rates between pre and post survey respondents and the inability to pair anonymized responses. Nonparametric testing *via* Mann-Whitney test was utilized as information was both ordinal and not normally distributed.

## Results

Of the approximately 40 attendees of the workshop, 18 residents completed the pre-session survey with 10 completing the post session survey. The average self-reported baseline experience in AI was 2.58/10 ± 0.6, with 1 being no experience whatsoever and 10 being expert level.

**Figure 3:**
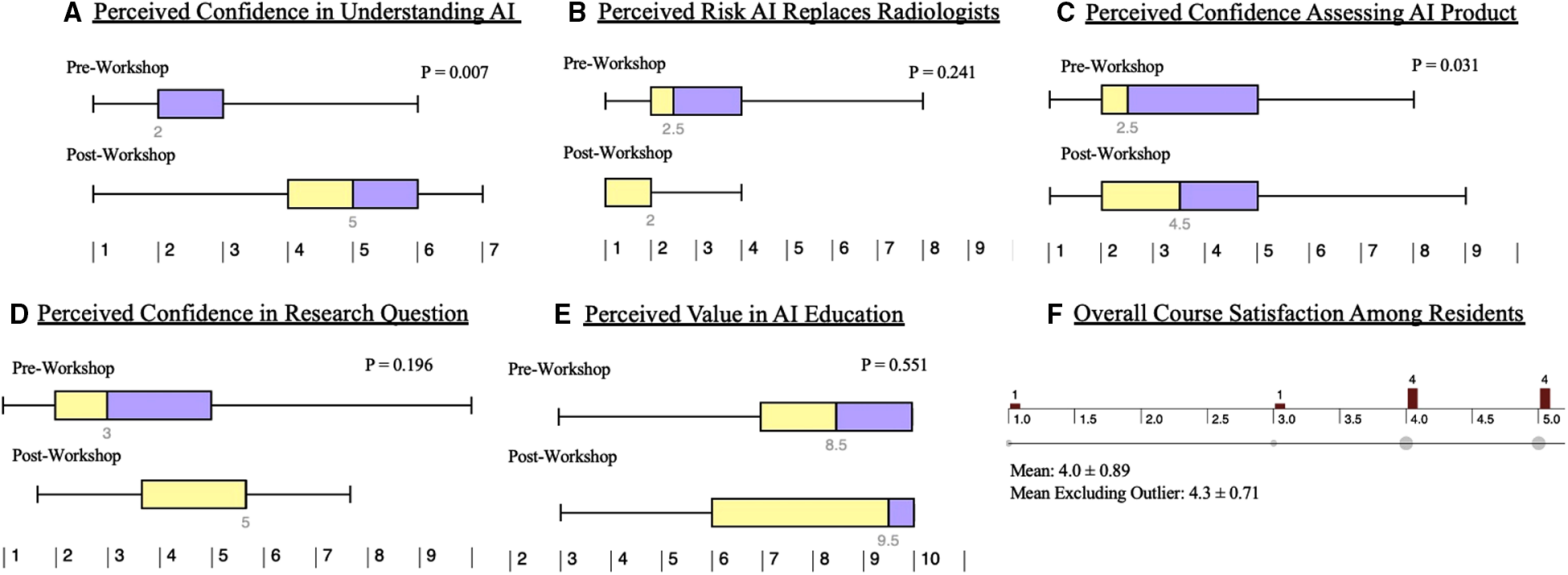
Learner perceptions of artificial intelligence before and after AI-RADS. The results of the pre and post workshop survey are summarized as above. There were significant increases in perceived confidence understanding artificial intelligence (**A**) as well as assessing artificial intelligence related products (**C**). Residents had a nonsignificant decrease in their perception of AI ultimately replacing radiologists (**B**). There was a non-statistically significant increase in perceived confidence in formulating an AI-related research question (**D**). The workshop garnered an average satisfaction rating of 4.0/5 (**F**); exclusion of the outlier resulted in an average of 4.3/5.

Perceived understanding of artificial intelligence in the context of reading a primary journal article was a primary end metric in both the original course and in the truncated version. In the longitudinal AI-RADS curriculum, six out of the seven lectures demonstrated statistically significant increases in self-reported perceived learner confidence, with the final lecture approaching significance at *P* = 0.07. This trend was redemonstrated in the one-day workshop (Figure 3A), with *P* = 0.007 by Mann-Whitney testing.

For the original AI-RADS course, baseline learner impressions of artificial intelligence were subjectively attained. This was adjusted in the workshop such that more objective qualitative data could be assessed. In the one-day workshop, learners did not demonstrate a statistically significant change in perceived likelihood of artificial intelligence replacing radiologists. In both the pre and post session surveys, the overall results signified low perceived likelihood (Figure 3B).

Learners demonstrated a statistically significant (*P* = 0.031) increase in confidence in their ability to assess new clinical applications of artificial intelligence over time (Figure 3C).

However, learners demonstrated a non-statistically significant increase in their ability to formulate research questions related to artificial intelligence (Figure 3D).

Learners did not demonstrate a significant change in perceived importance of AI education (Figure 3E).

Finally, learners maintained an overall average high perception of the overall quality of the workshop. Post-session response distribution (*n* = 10) can be seen in Figure 3F, with an average response of 4.0 ( ± 0.89) out of 5. Exclusion of the outlier yields a mean of 4.33 ( ± 0.71).

Data collected from attending physicians was sparse and is not included due to significant attrition between the pre and post survey respondents. On average, attendings rated the perceived importance of artificial intelligence education as 8.6 ± 1.4, while residents rated it as 7.85 ± 1 (*P* = 0.138).

## Discussion

In both the longitudinal curriculum and in the one-day workshop, the AI-RADS curriculum was well received by learners, garnering a 9.8/10 and a 4.3/5 (excluding singular outlier), respectively. This high metric of overall satisfaction is encouraging, especially coupled with the statistically significant increase in confidence in perceived understanding of artificial intelligence as well as perceived confidence in assessing new clinical products. Though there was no statistically significant change observed in perception of the importance of AI education in radiology, median perceived importance remained high at 8.5/10 prior to the conference vs. 9.5/10 thereafter, suggesting sustained if not intervally increased interest in the field.

This one-day workshop did demonstrate several limitations. While in broad strokes, learners were more confident in their understanding of artificial intelligence, their perceived confidence in formulating their own research questions related to artificial intelligence was increased but not significantly so. This may be related to a number of factors including the baseline expedited nature of content delivery in the setting of a one-day workshop, the relatively high degree of resident attrition between the beginning and end of the session (18 starting, 10 finishing), the presence of obvious outliers, and the relatively small sample size. While the final integrative activity was intended to inspire confidence on this front, in reality there are rarely concrete situations where one algorithm clearly is better than others: much of machine learning is exhaustive experimentation. Importantly, however, the concepts alluded to by this question require significant higher order thinking. In addition to the above-mentioned limitations in data collection, it is very possible that the lack of statistical significance is reflective of the difficulty and complexity inherent to this task and the concentrated method by which content was delivered.

Assessment of the net impact of these results are limited, as surveys were administered immediately before and after the workshop. Longitudinal follow-up surveys may provide insight in long term retention, changes in perception, or changes in practice (i.e., AI utilization, research projects started, more advanced coursework pursued, etc.). Furthermore, these results are the product of an internal evaluation without a matched comparison group.

The workshop itself was attended by approximately 40 attendees. Some of these attendees arrived late and left early, thus not completing the pre and post surveys. This is particularly evident in the attendees who were attending physicians, whose data is not displayed due to the extremely high rate of attrition (either by leaving early or by failing to complete the survey), rendering their survey results unusable for analysis. This may be reflective of a combination of the high clinical burdens placed on attending radiologists, limits in academic time compared to trainees, or other factors. Interestingly, attendings rated the importance of AI education higher (although not statistically significantly so) as compared to residents.

Additional incentives besides offering CME credit may improve engagement and retention, such as institutions offering protected time for AI education, creating financial incentives for attendings who participate in similar events, or providing academic certifications for those who complete a certain number of courses. More intensive options could include tying AI education to faculty promotion or institutional accreditation. More broadly, while AI is widely considered to be a topic of high importance within the radiology community, as of writing there is no concretely defined Accreditation Council for Medical Education (ACGME) requirement for diagnostic radiology residency programs to include artificial intelligence within their training programs or inclusion of AI within the American Board of Radiology (ABR) Core exam; this implicitly devalues the relative importance of AI education and, in the finite hours of residency training, places seeking further education in AI at the opportunity cost of learning other material.

At time of creation, both curricula were not designed with specific instructional pedagogies in mind. While both utilized direct learner participation through the original AI-RADS monthly journal club and the workshop's final integrative activity, they were fundamentally based on older didactic-based methods of content delivery. Though the decision to host the workshop virtually was based on pandemic-related logistical constraints, at least some future deliveries of this workshop will be virtual given the obvious convenience and broader acceptance as a learning platform. Effective practices for adult online learning include several techniques, including relationship/community building among attendees, incorporating active learning activities, embracing learner agency, and personalization (9). While the inherent structure of a single-day workshop limits the ability to address some of these domains, there is room to restructure the course to allow greater learner choice (e.g., à la carte topics, choice of research problems, etc.) and audience participation/interaction (e.g., use of break-out rooms, think-pair-share breakout rooms, etc.). In addition to the above, future iterations of the course include a transition from subjective self-reported metrics of understanding to more objective content related quizzes as well as the inclusion of additional open-ended free text responses which could be mined to better understand barriers for adoption, attitudes, and beliefs.

Despite these limitations, this unique one-day workshop for radiologists in training with little to no computer science background demonstrated promising results. While the entirety of the workshop was offered for CME credit, shorter versions of individual lectures can be found online at https://pages.acr.org/Informatics-e-learning-hub-ai-for-the-practicing-radiologist.html.

The landscape of artificial intelligence in clinical imaging today demands for radiologists familiar with these techniques in the near tomorrow. Indeed, it is not unrealistic to anticipate core concepts in machine learning to become a fundamental aspect of radiologist training analogous to magnetic resonance physics. There is both significant want and pragmatic need for radiologists to understand these techniques. The promising results of both the longitudinal AI-RADS curriculum as well as the condensed single-day version are suggestive of a potential new way forward in engaging trainees with this material.

## Data Availability

The raw data supporting the conclusions of this article will be made available by the authors, without undue reservation.

## Appendix A Syllabus

**COURSE TITLE: *Artificial Intelligence for the Practicing Radiologist***

**Target Audience:**

(1) Radiology trainees: residents and fellows
(2) Practicing radiologists without training in computer science or data science

**Specific Learning Objectives:**

After completing this course, attendees should be able to:

(1) Recognize 6 foundational concepts in machine learning and their resultant algorithms
(2) Describe the strengths and weaknesses of each algorithm
(3) Describe commonly used metrics to evaluate the performance of algorithms
(4) Apply these concepts to real-life design problems

**Goals in narrative form:**

The proposed one-day course foundational concepts in artificial intelligence algorithms and their application to problems or tasks in radiology.

This course's approach to teaching artificial intelligence is analogous to an approach one might take teaching MRI physics to radiologists who aren't trained physicists or engineers. While all radiologists need to have some basic conceptual understanding of the physics behind common MR sequences, the vast majority of radiologists functionally only need to recognize the utility and limitations of different sequences in assessing specific pathology.

Similarly, though most practicing radiologists will not become AI researchers themselves, they will need to understand the basic strengths/limitations of these techniques in a clinical context. The goal for this course is to familiarize attendees with a broad conceptual understanding so that in the near-future, when multiple different AI applications are part of the everyday workflow, the radiologist has a better sense when to “trust” the algorithm output and identify when the algorithm is appropriately or inappropriately applied.

**Course outline:**

1) **Computational basics [15 min]**
  a) Course introduction
  b) Word cloud on perceptions of AI
    i) Brief (5 min) discussion of common themes (e.g. “replacement,” etc.)
  (c) Learning objectives:
    i) Define “algorithm,” “features,” “GIGO.”
    ii) Delineate artificial intelligence, machine learning, and neural networks
2) **Naive bayes [30 min]**
  a) Probability review
    i) Conditional probability nomenclature
    ii) Conceptualizing dependency
  b) The spam filter problem
  c) Feature selection
    i) What makes for a good feature?
    ii) How can features be used in tandem
  d) The Naive Bayes Algorithm
  e) On Bias and Limitations
  f) Naive Bayes in radiology
    i) Al Assad et al “Application of machine learning to evaluate the adequacy of information in radiology orders.”
3) **K-Nearest Neighbors & Principal Component Analysis [45 min]**
  a) Data visualization and feature selection revisited
  b) The Netflix Problem
    i) Categorization of complex data
    ii) Information as topology
  c) Nearest Neighbor approach to data similarity
  d) Introduction to the Curse of Dimensionality
    i) More features != better
    ii) Relative “thinness” of information density
  e) Consolidation and principal component analysis
    i) Introduction to data processing
    ii) Feature reduction and variance
  f) Limitations
  g) Applications of KNN + PCA in radiology
    i) Li et al “Using the K-NN algorithm for the classification of lymph node metastasis in gastric cancer.”
4) **Ensembles [45 min]**
  a) Data types, revisited
    i) Categorical vs. numeric vs. ordinal
  b) Parsing mixed data
    i) Introduction to decision trees
  c) The Sepsis Protocolling Problem
    i) Integration of mixed data to categorize sepsis risk
    ii) Complex entity that requires multiple streams of information to detect
  d) The Random Forest
    i) Introduction to ensembles
    ii) Introduction to bootstrapping/synthetic data
    iii) RF parameters
  e) Error and data purity
    i) Introduction to Gini Impurity
    ii) Introduction to error tolerance
  f) Gradient Boosting
    i) Alternative but related concept to RF
  g) Curse of Dimensionality Revisited
  h) Limitations
  i) Applications of Ensembles in Radiology
    i) Carrodeguas et al “Use of machine learning to identify follow-up recommendations in radiology reports.”
5) **Linear Classifiers [45 min]**
  a) Early machine vision: a naval adventure
    i) The image detection problem
    ii) Machine representation of images
  b) Data visualization, revisited (hyperplanes and high dimensional partitions)
  c) Linear separation: The perceptron
    i) Linear manipulation
    ii) Kerneling
  d) Support vector machines
    i) Tolerance and decision boundaries
    ii) Introduction to error types
  e) Curse of Dimensionality, Revisited
  f) Limitations
  g) Applications of SVM in Radiology
    i) Liu et al “Prediction of hematoma expansion in spontaneous intracerebral hemorrhage using support vector machines.”
  h) From the depths: return to early machine vision
6) **Neural Networks [45 min]**
  a) Data visualization, revisited
  b) Nuanced boundaries: the multilayer perceptron
  c) Image representation revisited
    i) Convolution and feature extraction
    ii) Piecewise image dissection
    iii) Masking and edge detection
  d) Convolutional Neural Networks
    i) Network architecture
    ii) Broad overview of capabilities
  e) Limitations
    i) Curse of dimensionality revisited
    ii) Where machines fail
  f) Applications of Neural Networks in Radiology
  g) AI and the future of Radiology, a student perspective
7) **Integrative Final Activity [60 min]**

## Appendix B Post-session Survey Form (Likert-10 unless otherwise specified)

What is your level of training? (Resident/Fellow/Attending/Other)

How would you rate this workshop overall?

How much experience do you have with AI?

How confident are you in your understanding of AI/ML algorithms?

How concerned are you about AI and the future of Radiology?

How likely do you think AI will eventually replace radiologists?

How important do you think AI education will be for the next generation of radiologists?

How confident do you feel formulating a research question that uses AI?

How confident do you feel assessing a new clinical product that employs aI?
